# Perfection of Perovskite Grain Boundary Passivation by Rhodium Incorporation for Efficient and Stable Solar Cells

**DOI:** 10.1007/s40820-020-00457-7

**Published:** 2020-06-03

**Authors:** Wei Liu, Nanjing Liu, Shilei Ji, Hongfeng Hua, Yuhui Ma, Ruiyuan Hu, Jian Zhang, Liang Chu, Xing’ao Li, Wei Huang

**Affiliations:** 1 Institute of Advanced Materials and New Energy Technology Engineering Laboratory of Jiangsu Province, Nanjing University of Posts and Telecommunications (NUPT), Nanjing, 210023 People’s Republic of China; 2 Shaanxi Institute of Flexible Electronics, Northwestern Polytechnical University, Xi’an, 710072 People’s Republic of China; 3 Guangdong Provincial Key Lab of Nano-Micro Materials Research, School of Chemical Biology and Biotechnology, Peking University Shenzhen Graduate School, Shenzhen, 518055 People’s Republic of China

**Keywords:** Perovskite solar cells, Grain boundary passivation, Rhodium incorporation

## Abstract

Organic cation and halide anion defects are omnipresent in the perovskite films, which will destroy perovskite electronic structure and downgrade the properties of devices. Defect passivation in halide perovskites is crucial to the application of solar cells. Herein, tiny amounts of trivalent rhodium ion incorporation can help the nucleation of perovskite grain and passivate the defects in the grain boundaries, which can improve efficiency and stability of perovskite solar cells. Through first-principle calculations, rhodium ion incorporation into the perovskite structure can induce ordered arrangement and tune bandgap. In experiment, rhodium ion incorporation with perovskite can contribute to preparing larger crystalline and uniform film, reducing trap-state density and enlarging charge carrier lifetime. After optimizing the content of 1% rhodium, the devices achieved an efficiency up to 20.71% without obvious hysteresis, from 19.09% of that pristine perovskite. In addition, the unencapsulated solar cells maintain 92% of its initial efficiency after 500 h in dry air. This work highlights the advantages of trivalent rhodium ion incorporation in the characteristics of perovskite solar cells, which will promote the future industrial application.

**Electronic supplementary material:**

The online version of this article (10.1007/s40820-020-00457-7) contains supplementary material, which is available to authorized users.

## Highlights

- Rhodium ion incorporation helps the nucleation of perovskite grain, passivates the defects in the grain boundaries and enhances the film quality, charge carrier lifetime and mobility.
- After optimizing 1% rhodium into perovskite film, the solar cells achieve an efficiency of 20.71% without obvious hysteresis.

**Electronic supplementary material:**

The online version of this article (10.1007/s40820-020-00457-7) contains supplementary material, which is available to authorized users.

## Graphical abstract

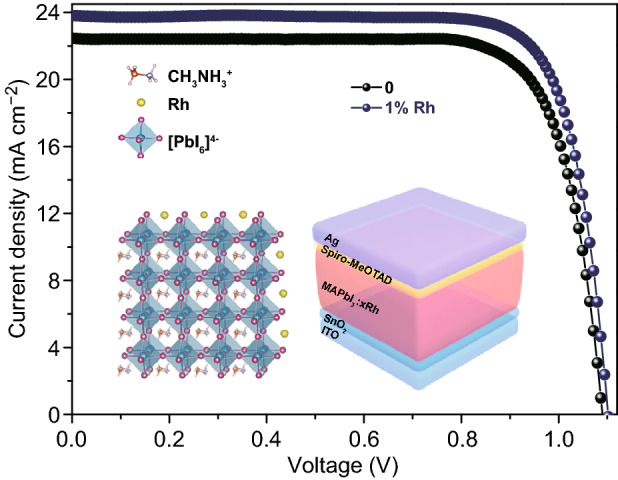

## Introduction

Halide perovskites have attracted great attention owing to the eminent optoelectronic properties, such as suitable energy bandgap [1–6], high absorption coefficient [7–9], long charge diffusion length [10–13] and high carrier mobility [14, 15]. Those advantages boost the improvement in perovskite solar cells (PSCs), being with certified power conversion efficiency (PCE) up to 25.2% [16].

However, pristine perovskite has inevitable internal defects, such as Pb and I vacancy defects, especially in gain boundary, which reduce the performance and stability of PSCs. To date, doping metal ion is an effective strategy to reduce defects, which can improve efficiency and stability of PSCs [17]. For example, doping with monovalent metal cations (Cu^+^, Ag^+^ or Li^+)^ was applied to reduce trap-state density [18, 19], improved perovskite crystallinity and film quality, thus enhanced performance of PSCs. By doping bivalent cations (Mn^2+^, Co^2+^ or Zn^2+^), tuned electronic band structures and crystalline morphology were received and improved the stability of PSCs [20–27]. Trivalent metal cations (In^3+^, Eu^3+^ and Al^3+^) were often used to decrease deep defects, optimize film morphology and increase efficiency and stability of PSCs [28–31]. Especially for Eu^3+^ doping, device achieves long-term durability with 92% of the original PCE under continuous illumination for 1500 h [31]. Hence, finding more trivalent metal ions to obtain excellent properties of PSCs is important. The radius of Rh^3+^ is 67 pm, which is less than Pb^2+^ (119 pm). A smaller radius allows both interstitial doping and the possibility of partial replacement of Pb^2+^. Moreover, 4*d* orbital of Rh^3+^ probably tunes electrical properties of perovskite films by heterovalent incorporation [32, 33]. Clearly, compared to elements doped without *d* orbitals (such as, Al^3+^), such method definitely advances hybrid perovskite materials [30]. Therefore, it is anticipated that Rh^3+^ can be used in hybrid perovskite to enhance the properties of devices.

Here, we utilized Rh^3+^ to be incorporated with MAPbI_3_ for developing MAPbI_3_:*x*Rh^3+^ (where *x* is excessive rhodium mol ratio, and *x* = 0, 0.5, 1.0 and 5.0 mol%). Rh^3+^ incorporation with tiny amounts can help the nucleation of perovskite grain and passivate the defects in perovskite film boundaries. In addition, 1% Rh^3+^ incorporation with perovskite films possesses larger crystalline and less pinhole, which leads to reduce trap-state density and enlarge charge carrier lifetime. Planar heterojunction PSCs with 1% Rh^3+^ incorporation exhibit high PCE of 20.71% and significantly suppressed photocurrent hysteresis. Compared to other studies of PSCs, the PSCs based on Rh^3+^ incorporation MAPbI_3_ achieved higher PCE (Table S11). Meanwhile, the devices of 1% Rh^3+^ incorporation have high stability within 500 h without encapsulation in dry air. This work highlights the advantages of Rh^3+^ incorporation in PSCs, which can promote the future industrial application.

## Experimental Section

### First-Principle Calculation

All of the density functional theory (DFT) calculations were employed by VASP code [34]. Generalized gradient approximation (GGA) of the projector augmented wave (PAW) was employed [35]. The plane-wave energy cutoff is 500 eV. The energy cutoff convergence is 1 × 10^−4^ eV, and the force cutoff convergence is − 0.09 eV Å^−1^ [36, 37]. For perovskite structure, 3 × 3×3 Monkhorst–Pack grid is taken [38]. The results of calculated lattice constants are well agreed with the experimental from Rietveld refinement (Table S2).

### Preparation of Perovskite Single Crystals

For MAPbI_3_ single crystal, PbI_2_ (2.835 g) and MAI (0.978 g) were mixed in 5 mL γ-butyrolactone under stirring at 70 °C for 2 h. After heating the solution at 150 °C for 24 h, some obvious single crystals were obtained, which were washed with γ-butyrolactone and dried at 50 °C. Select a relatively large single crystal as the seed crystal, the above reaction was repeated once [11]. For MAPbI_3_:*x*Rh (where *x* = 0.5, 1 and 5%) single crystal, *x* is excessive RhI_3_ incorporation, and the mixed PbI_2_ and MAI solution was added 0.5, 1 and 5 mol% (0.015, 0.030 and 0.149 g) RhI_3_, respectively.

### Preparation of Devices

SnO_2_ dense layer (2.67%, diluted by deionized water) was prepared for the cleaned ITO substrate by spin-coated method. For MAPbI_3_:*x*Rh (where *x* = 0, 0.5, 1, 5%) solution, 159 mg MAI, 470 mg PbI_2_ and 0, 0.5, 1 and 5 mol % (0, 2.42, 4.84 and 24.2 mg) RhI_3_ were separately dissolved in 0.8 mL DMSO:DMF (1:4) solution under room temperature. In total, 35 μL perovskite solution dipped on the SnO_2_ layers and then spun at 4000 rpm for 20 s. In total, 300 μL chlorobenzene was dipped on the substrate to enhance the film quality when the spinning at 10 s. Then, the substrate was annealed at hot plate. The hole transporting layer Spiro-OMeTAD was spin coated as our previous work [21]. Finally, the PSCs were evaporated 150 nm Ag (0.8 Å s^−1^) with area of 0.09 cm^2^.

## Results and Discussion

**Fig. 1:**
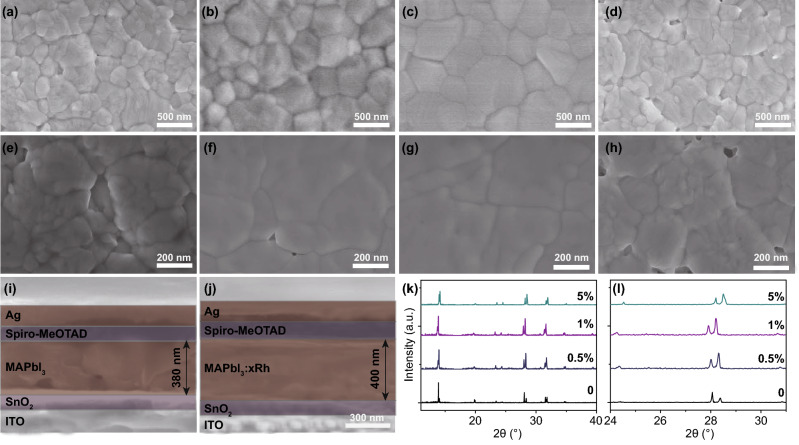
SEM pictures of MAPbI_3_:*x*Rh [where *x *= 0 (**a**, **e**), 0.5% (**b**, **f**), 1% (**c**, **g**) and 5% (**d**, **h**)] films. **i**, **j** Cross-sectional pictures of PSCs fabricated by MAPbI_3_:*x*Rh (where *x *= 0, 1%). **k**, **l** XRD patterns of the MAPbI_3_:*x*Rh single crystal (where *x *= 0, 0.5%, 1% and 5%)

**Fig. 2:**
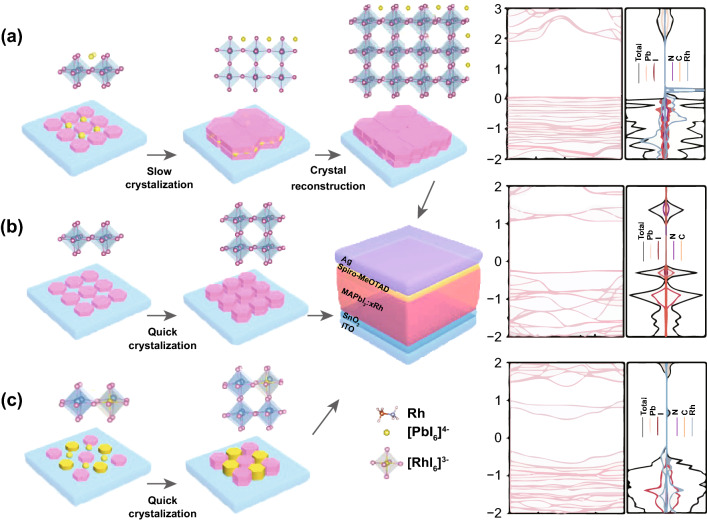
Schematic plot of mechanism for Rh^3+^-induced perovskite crystallization. **a** 1% Rh^3+^-incorporated in MAPbI_3_ through Rh-N band and Rh-I band to form large crystalline structure. **b** Pristine MAPbI_3_ film formed by quick crystallization. **c** 5% Rh^3+^-incorporated in MAPbI_3_ film. The diagram on the right is the corresponding band structures and density of states from the first principles (**a** interstitial 6% Rh incorporation, **b** MAPbI_3_, **c** MAPb_0.875_Rh_0.125_I_3_)

The characteristics of device are closely related to the morphology of the absorption film. Figure 1 shows top-view SEM pictures of MAPbI_3_:*x*Rh (*x* = 0, 0.5%, 1% and 5%) (*x* represents Rh excessive incorporation). At low Rh^3+^-incorporated concentration, such as *x* = 0.5% and 1%, the grain size becomes larger than pristine MAPbI_3_ film without Rh^3+^-incorporated (Fig. 1f, g). However, at high Rh^3+^-incorporated concentration (where *x* = 5%), the quality of film becomes worse (Fig. 1h). The phenomenon was explained both theoretically and experimentally. From the experimental perspective, when the Rh^3+^-incorporated concentration is 0.5% and 1%, a small quantity of Rh^3+^ aggregated near the octahedral [PbI_6_]^4−^, decreasing the process of perovskite crystallization, organizing large crystalline and uniform perovskite films (Fig. 2a). However, when the Rh^3+^-incorporated concentration is over 5%, on the one hand, a large number of excessive Rh^3+^ aggregate at octahedral [PbI_6_]^4−^ and prevent the crystallization of perovskite, resulting in the poor-quality film; on the other hand, both Rh^3+^ and Pb^2+^ may be acted as the nuclei to quick crystallization and form discontinuous perovskite films (Fig. 2c) [24]. Thus, when perovskite thin films are based on MAPbI_3_:*x*Rh (where *x* = 5%), the film appears pinhole (Fig. 1h). The reason for this change in perovskite films is the different speed of film formation (Fig. 2). From a theoretical point of view, when the Rh^3+^-incorporated concentration of Rh^3+^ is 1%, Rh^3+^ through chemical bonds with the surrounding [PbI_6_]^4−^ is to induce ordered arrangement and reduce defects [18]. However, when the Rh^3+^-incorporated concentration of Rh^3+^ is up to 5%, partial Rh^3+^ may replace Pb^2+^ to form additional perovskite structures. From intersecting surface SEM pictures of PSCs fabricated by MAPbI_3_ and MAPbI_3_:*x*Rh (where *x* = 1%), there is no pinhole in the cross section after Rh^3+^ incorporation. This shows that Rh^3+^ incorporation makes both plane and cross section of perovskite continuous [39]. The film thicknesses with its deviations from (where *x* = 0, 1%) layers are 380 and 400 nm. Electron energy loss spectroscopy (EELS) mapping analysis of MAPbI_3_:*x*Rh (where *x* = 1%) shows that the atomic % is also similar to the ratio of experimental preparation (Table S1). From EELS mapping, most of rhodium atoms are distributed at the grain boundary. By grain boundary passivation of rhodium ions, larger perovskite grains without pinholes film were formed (Fig. 1g). In order to explore physical mechanism of MAPbI_3_:*x*Rh materials, we prepared the MAPbI_3_:*x*Rh (where *x* = 0, 0.5%, 1% and 5%) single crystal (Fig. S3). From Fig. 1k, l, XRD was measured to research the crystallinity of MAPbI_3_:*x*Rh single crystalline. The peaks of 14° and 28° that correspond to the (101) and (202) lattice planes can be clearly seen from XRD, which is indicated that the MAPbI_3_:*x*Rh possesses excellent crystallinity. No other peaks are observed for the MAPbI_3_:*x*Rh perovskites, which are indicated that the Rh^3+^ and Pb^2+^ cations have not formed different kinds of phases. Moreover, the diffraction angles are slight lessening when it is 0.5% Rh^3+^ incorporation MAPbI_3_ (Fig. 1k). The reason of that is the unit cell of MAPbI_3_:*x*Rh (where *x* = 0.5%) was enlarging from Rietveld refinement result (Table S2). However, when there is excessive 5% Rh^3+^ incorporation, the peak shift to a higher diffraction angle indicates the lattice parameters decrease. Maybe smaller Rh^3+^ cations partially displace the larger Pb^2+^ cations and the cell volume decreases (Table S2).

We set up the corresponding model and study electronic and optical properties of MAPbI_3_:*x*Rh (where *x* = 0, 6%, 8% and 12.5%) and MAPb_0.875_Rh_0.125_I_3_ (Figs. 2 and S1, S5) based on the result of XRD Rietveld refinement. In perovskite system, iodide ion and methylamine ion are easy to dissociate, which is destroyed perovskite structure and resulted in iodine vacancy defect and methylamine ion vacancy defect. From first-principle calculation, the binding energy (formula as *E *=* E*_doped_ − *E*_bulk_ − *E*_doping atom_) is − 3.45 eV [40], indicating that Rh^3+^ can easily insert into the interstices of perovskite through the chemical bonds with iodine ion to enhance the stability of perovskite structure and prevent the ions from escaping and reduce iodine vacancy defects. Therefore, the crystallization of perovskite, tiny amount of Rh^3+^ is near the octahedral [PbI_6_]^4−^ to induce ordered arrangement of organic cations to form high-quality film with few defects (Fig. 2a). From first-principle calculation, the bandgap values of MAPbI_3_:*x*Rh (where *x* = 0, 6%, 8% and 12.5%) and MAPb_0.875_Rh_0.125_I_3_ are 1.31, 1.84, 1.14, 0.80 and 1.15 eV, respectively. The calculated bandgap of 1.31 is similar to experimental bandgap of 1.57 eV. There is a small error in the results of experiment and calculation. For the density of states (DOS) of MAPbI_3_, valence band maximum (VBM) and conduction band minimum (CBM) are mainly affected by orbitals hybridization of I and Pb. When rhodium ions are interatrial in perovskite structure, hybridization between rhodium and other atomic orbitals affects the distribution of DOS. From Fig. 2a, the rhodium atom makes the conduction band minimum move toward a higher energy level, thus increasing the bandgap. In Fig. 2b, DOS and band structure shows that the MAPbI_3_ are nonmagnetic. From Fig. 2a, the spin-up and spin-down band structure is not imperfect symmetry, suggesting MAPbI_3_:*x*Rh possesses magnetism. The calculated total magnetic moment of MAPbI_3_:*x*Rh (where *x* = 6%) atom is 2.254 μB mainly attributed to Rh atom. This indicates the potential application of this material in the field of magnetism.

**Fig. 3:**
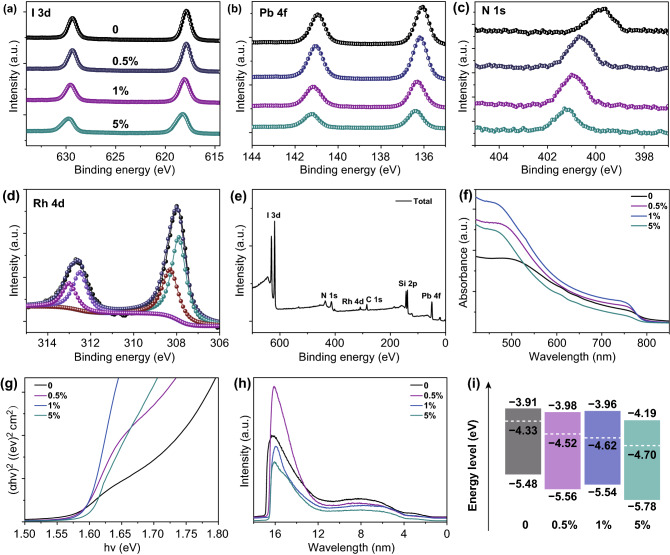
XPS of **a** I 3*d*, **b** Pb 4*f*, **c** N 1*s* from MAPbI_3_:*x*Rh (where *x *= 0, 0.5%, 1% and 5%). **d** Rh 4*d* from MAPbI_3_:*x*Rh (where *x *= 1%). **e** XPS of total elements from the MAPbI_3_:*x*Rh (where *x *= 1%). **f** Absorbance coefficient of MAPbI_3_:*x*Rh. **g** Estimated bandgap potential of perovskite films. **h** UPS of MAPbI_3_:*x*Rh (where *x *= 0, 0.5%, 1% and 5%). **i** Energy level diagram of MAPbI_3_:*x*Rh (where *x *= 0, 0.5%, 1% and 5%)

XPS was also further to verify Rh^3+^ incorporation and study chemical bonding states of the MAPbI_3_:*x*Rh structure. Figure 3a shows a separation of approximately 11.6 eV from I 3*d*_3/2_and 3*d*_5/2_ spectra. As the Rh concentration increased, the binding energy (BE) of I 3*d* is slightly toward higher. From Fig. 3b, the BE of the Pb 4*f*_7/2_ was about 136.5 eV. As the Rh concentration is increased, the BE of Pb 4*f* is also slightly toward higher. This small transition to high binding energy may be the shorter Rh–I distance than Pb–I distance, which is lead to higher energy of Pb(Rh)–I bond. We can also observe from the Rietveld refinement above (Table S2). In addition, as the Rh^3+^-incorporated concentration increases, the 1*s* orbital of N also moves to a higher binding energy. Stronger interactions of Rh–I bond can reduce iodine vacancy defects. The peak at 315 and 308 eV of MAPbI_3_:*x*Rh (where *x *= 1%) film indicates the presence of Rh elements (Fig. 3d).

In order to study the distribution of energy level, absorbance coefficient and ultraviolet photoelectron spectroscopy (UPS) spectra were tested. When the Rh^3+^-incorporated concentration increased from 0 to 1%, the intensity of the absorption spectrum also increased. However, absorption intensity of 5% Rh^3+^ incorporation decreases, which may be related to the quality deterioration of perovskite films. We also calculated the absorption spectra, which were generally consistent with the experiment (Fig. S5a). The bandgap of MAPbI_3_:*x*Rh is calculated by converting the UV/Vis absorption spectrum into Tauc plots (Fig. 3g). On the basis of the Kubelka–Munk theory [21], the bandgap of MAPbI_3_:*x*Rh (where *x* = 0, 0.5%, 1% and 5%) is determined to be 1.570, 1.58, 1.58 and 1.59 eV, respectively. From Figs. 3f and S5a, with the increase of Rh^3+^-incorporated concentration, the absorption range was slightly blue shift and basically consistent with the increase of bandgap. Figure 3h shows UPS image of MAPbI_3_:*x*Rh (where *x* = 0, 0.5%, 1% and 5%). The specific energy levels were calculated from UPS and UV/Vis absorption (Fig. 3i). The formula for calculating the Fermi energy level is *E*_F_ = 21.22 − *E*_B_ [21] (where *E*_F_ is the Fermi level, and *E*_B_ is high binding energy cutoff). The high binding energy cutoff of MAPbI_3_ is 16.89 eV. *E*_F_ of MAPbI_3_ is − 4.33 eV (21.22–16.89). The VBM of MAPbI_3_ is the *E*_F_ minus low binding energy as − 5.48 eV (− 4.33–1.15). The CBM of MAPbI_3_ is the sum of VBM and bandgap as − 3.91 eV. Other detail energy levels of MAPbI_3_:*x*Rh can be calculated in this way. Energy level diagram of MAPbI_3_:*x*Rh (where *x *= 0, 0.5%, 1% and 5%) is shown in Fig. 3i. The detailed analysis of energy levels is shown in Table S3.

**Fig. 4:**
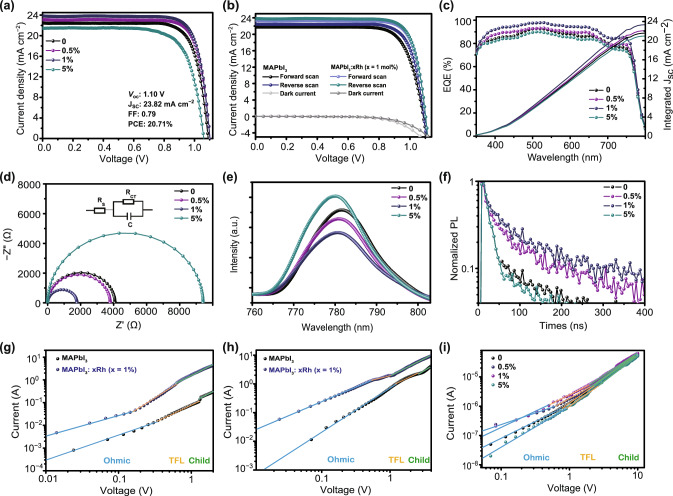
**a** *J*–*V*, **c** EQE data and **d** Nyquist plots of PSCs fabricated by MAPbI_3_:*x*Rh (where *x *= 0, 0.5%, 1% and 5%). **b** Forward and reverse direction *J*–*V* of PSC fabricated by MAPbI_3_:*x*Rh (where *x *= 0, 1%). **e** PL spectra of the MAPbI_3_:*x*Rh/SnO_2_ films. **f** Time-resolved PL spectrum of perovskite films based on MAPbI_3_:*x*Rh (where *x *= 0, 0.5%, 1% and 5%). *I*–*V* characteristic of **g** hole-only device, **h** electron-only device prepared by MAPbI_3_ and MAPbI_3_:*x*Rh (*x* = 1%). **i** Perovskite-only device fabricated by MAPbI_3_:*x*Rh (where *x *= 0, 0.5%, 1% and 5%)

The current density versus voltage (*J*–*V*) curve is important to study photovoltaic properties. *J*–*V* curves of PSCs are fabricated by structure of ITO/SnO_2_/MAPbI_3_:*x*Rh/Spiro-MeOTAD/Ag (Fig. 4a). Device photovoltaic parameters with MAPbI_3_:*x*Rh are shown in detail (Table 1). PSCs fabricated by pristine perovskite possess short-circuit current density (*J*_sc_) of 22.46 mA cm^−2^, open-circuit voltage (*V*_oc_) of 1.09 V, fill factor (FF) of 0.78, PCE of 19.09%. The PSCs prepared by MAPbI_3_:*x*Rh (where *x* = 1%) possess high PCE of 20.71% (Table 1), which is increased approximately 10% compared to that of based on MAPbI_3_. However, the properties of PSCs based on MAPbI_3_:*x*Rh (where *x* = 5%) have decreased. The MAPbI_3_:*x*Rh (where *x* = 5%) films possess poor-quality films, which leads to large leakage current, low *J*_sc_. To ensure repeatability of device performance, over 40 PSCs are fabricated and characterized (Tables S5–S8). PSCs based on MAPbI_3_ show a wide photoresponse in the range of 350-800 nm, and external quantum efficiency (EQE) values were close to 85%. EQE values of devices fabricated by 1% Rh incorporation are risen to more than 90%. The photocurrent of 21.62, 22.13, 23.37 and 21.00 mA cm^−2^ is obtained by integration of EQE spectrum in the range of 350–800 nm, which is similar to the *J*_sc_ from the results of *J*–*V* measurement.

**Table 1:** Device photovoltaic parameters of devices based on MAPbI_3_:*x*Rh (where *x *= 0, 0.5%, 1% and 5%)

Devices		*V*_oc_ (V)	*J*_sc_ (mA cm^−2^)	FF	PCE (%)
MAPbI_3_	Best	1.09	22.46	0.78	19.09
	Average	1.07 ± 0.02	22.60 ± 0.8	0.75 ± 0.04	18.19 ± 0.96
0.5% Rh	Best	1.09	23.13	0.79	20.01
	Average	1.08 ± 0.02	22.72 ± 0.43	0.78 ± 0.03	19.37 ± 1.02
1% Rh	Best	1.10	23.82	0.79	20.71
	Average	1.10 ± 0.03	23.47 ± 0.41	0.76 ± 0.05	19.56 ± 1.01
5% Rh	Best	1.06	21.44	0.73	16.76
	Average	1.03 ± 0.03	21.30 ± 1.06	0.67 ± 0.06	14.98 ± 1.78

Charge transfer characteristics of PSCs were studied by electrochemical impedance spectroscopy (EIS). The EIS was tested in the dark under applied bias of V_OC_. Under such a condition, the charge carrier recombination resistance (*R*_REC_) attained the lowest value (*R*_REC_ ≪ *R*_CT_), and *R*_CT_ is the charge transfer resistance. Semicircle from EIS measurement is formed by series resistor (*R*_s_), charge transport resistor (*R*_ct_) and capacitor (CPE1). The inset of Fig. 4d is equivalent circuit diagram [41]. Because all devices have the same structure, *R*_S_ values are basically the same (Fig. S6). The radius of the arc represents the value level of *R*_ct_. The lowest *R*_ct_ of MAPbI_3_:*x*Rh (*x* = 1%) reveals that a tiny amount of Rh-incorporated can enhance charge transport capacity. Therefore, supreme *J*_sc_ is obtained from PSCs based on MAPbI_3_:*x*Rh (*x* = 1%). Figure 4e presents steady-state photoluminescence (PL) spectrum of MAPbI_3_:*x*Rh/SnO_2_ films. Excitation wavelength is 465 nm. For MAPbI_3_ films, a peak was around 780 nm. With the increase of Rh-incorporated content, the PL peak to appear slight blue shift. From Fig. 4e, the lower intensity of MAPbI_3_:*x*Rh (where *x* = 0.5%, 1%) film indicated extract electron carriers more effectively to SnO_2_ electron transport layer, which is consistent with the larger FF.

The device measured in the forward and reverse scanning directions. Hysteresis characteristics of photocurrent are analyzed by the results of *J*–*V* curves. Photocurrent hysteresis can be expressed by photocurrent hysteresis index (HI). The formula is (Eq. 1) [28]:

1 $${\text{HI}} = \frac{{{\text{PCE}}_{\text{reverse}} - {\text{PCE}}_{\text{forward}} }}{{{\text{PCE}}_{\text{reverse}} }}$$

The HI of PSCs fabricated by MAPbI_3_:*x*Rh (*x* = 0, 1%) is 0.042 and 0.020. Previous reports have shown that photocurrent hysteresis of devices mainly came from trap-induced carrier prevention. Therefore, lower photocurrent hysteresis indexes of devices fabricated by MAPbI_3_:*x*Rh (*x* = 1%) suggest that higher trap-induced carrier prevention is taken.

The time-resolved PL spectrums of MAPbI_3_:*x*Rh films are shown in Fig. 4f. Usually, *τ*_1_ is attributed to bimolecular recombination of photogenerated carriers, while *τ*_2_ is due to trap-assisted recombination. Table S4 shows the details of parameters of carrier lifetimes. The decay time of pristine perovskite is *τ*_1_ = 67 ns and *τ*_2_ = 693 ns. The decay time of MAPbI_3_:*x*Rh (*x* = 1%) is *τ*_1_ = 63 ns and *τ*_2_ = 818 ns. The passivation of rhodium ion mainly reduces grain boundaries and defects and increases carrier lifetime. To find the difference in average carrier lifetime (*τ*_avg_), the formula was defined as follows (Eq. 2) [42]:

2 $$\tau_{\text{avg}} = \frac{{\varSigma A_{i} \tau_{i}^{2} }}{{\varSigma A_{i} \tau_{i} }}$$

The *τ*_avg_ of pristine MAPbI_3_ film is only 671 ns. MAPbI_3_:*x*Rh (where *x* = 0.5%, 1%) films possess longer *τ*_avg_, which is 726 and 796 ns. Because there is no charge transport layer, nonradiative recombination is the main reason for decay lifetime. Rh^3+^ incorporation has long lifetime, which is reduced recombination and improve photocurrent of PSCs.

Space charge limited current (SCLC) was measured to study the mobility and defects density of perovskite films. Figure S7 shows device structures of the hole-only diode and the electron-only diode are shown. For electron-only device, SnO_2_ and PCBM layer are used as the electron transport (blocking holes) layer being coated on both sides of the perovskite. For the hole-only device, the NiO and Spiro-MeOTAD layer is utilized as hole transport (blocking electrons) layer being coated on both sides of the perovskite. The current versus voltage (*I*–*V*) was tested under dark conditions by a Keithley model 2400. Three regions were evident in the experimental data. *I*–*V* characteristics show three different regions: a linear ohmic region at low voltage (represented by the blue line); a trap-filling region from mediate voltage to the trap-filled limit voltage (*V*_TFL_) (represented by the orange line); a Child’s region (represented by the green line). The formula for trap density is [28]:

3 $$n_{t} = \frac{{2\varepsilon \varepsilon_{0} V_{\text{TFL}} }}{{eL^{2} }}$$

(where *V* is the relative dielectric constant of perovskite hybrid materials, *ε*_0_ is vacuum permittivity, *L* is the thickness of perovskite layer). The charge carrier mobility (*μ*) is estimated at the quadratic dependence region. The Mott–Gurney’s law is (Eq. 4) [28]:

4 $$I_{\text{d}} = \frac{{9\varepsilon \varepsilon_{0} \mu V^{2} }}{{8L^{3} }}$$

(where *I*_d_ is dark current density, and *V* is the applied voltage). The trap-filling process for the hole-only device set is at *V*_TFL_ = 0.33 V for MAPbI_3_ and *V*_TFL_ = 0.14 V for MAPbI_3_:*x*Rh (where *x* = 1%); the trap-filling process for the electron-only device set is at *V*_TFL_ = 1.12 V for MAPbI_3_ and *V*_TFL_ = 0.57 V for MAPbI_3_:*x*Rh (where *x* = 1%). As shown in Table S10, the hole trap density of 1% Rh^3+^-incorporated is 3.10 × 10^15^ cm^−3^ lower than MAPbI_3_ (8.09 × 10^15^ cm^−3^). The hole mobility of MAPbI_3_:*x*Rh (where *x* = 0, 1%) is 1.29 × 10^−3^ and 2.51 × 10^−2^ cm^2^ V^−1^ s^−1^. Similarly, the electron trap density of 1% Rh^3+^-incorporated is 1.26 × 10^15^ cm^−3^ smaller than MAPbI_3_ (2.74 × 10^16^ cm^−3^). The electron mobility of MAPbI_3_:*x*Rh (where *x* = 0, 1%) is 5.38 × 10^−3^ and 1.26 × 10^−2^ cm^2^ V^−1^ s^−1^. The decrease of trap densities of MAPbI_3_:*x*Rh (where *x* = 1%) leads to both electron and hole mobility increase. We also measured *I*–*V* curves of perovskite-only device by MAPbI_3_:*x*Rh (where *x* = 0, 0.5%, 1% and 5%). Because the trap density is proportional to *V*_TFL_, the trap density of MAPbI_3_:*x*Rh (where *x* = 1%) is minimum (Fig. 4i). In polycrystalline perovskite films, there are many defects such as vacancies, dislocations and bond deformation because of the confusion of atomic arrangement on grain boundaries. Defects are mainly distributed on the gain boundaries [43]. The results of defect density show that rhodium ion incorporation mainly plays the role of passivation grain boundary.

**Fig. 5:**
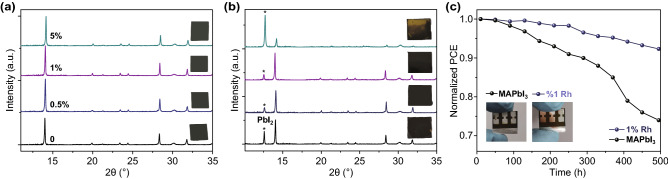
**a**, **b** XRD of perovskite films based on MAPbI_3_ and 1% Rh^3+^-incorporated is exposed in the air before and after two months. **c** PCE changes of perovskite solar cells located in dry air without encapsulation for 500 h

XRD patterns of MAPbI_3_ and 1% Rh-incorporated perovskite films are exposed in the humid air before and after two months (Fig. 5). The aging rate of perovskite films is related to PbI_2_ separated. Peak value of PbI_2_ is higher, and perovskite film aging is faster. From Fig. 5b, the perovskite films based on MAPbI_3_:*x*Rh (where *x* = 0.5% and 1%) aged slower than that based on MAPbI_3_ films. The addition of Rh^3+^ made the perovskite structure more stable once again. The perovskite film stability directly affects the PSCs stability. The PSCs were stored in dry atmosphere without encapsulation for 500 h (Fig. 5c). Figure 5c shows that PSCs based on MAPbI_3_:*x*Rh (where *x* = 1%) possess higher stability than that of MAPbI_3_, maintained 92% of initial PCE after 500 h. Detail performance of PSCs is fabricated by MAPbI_3_ and MAPbI_3_:*x*Rh with different aged time at dry air (Fig. S11 and Table S12).

## Conclusion

Herein, Rh^3+^ incorporation with tiny amount can help nucleation of perovskite grain, passivate grain boundary defects and improve properties of PSCs. In addition, Rh^3+^ incorporation with perovskite can contribute to preparing larger crystalline and uniform film, reducing trap-state density and enlarging charge carrier lifetime. Therefore, planar heterojunction PSCs by MAPbI_3_:*x*Rh^3+^ perovskite materials possess PCE of 20.71% without obvious photocurrent hysteresis. Meanwhile, the devices of Rh^3+^-incorporated have high stability within 500 h without encapsulation in dry air. This work highlights the advantages of Rh^3+^ incorporation in the capabilities of PSCs, which will promote the future industrial application.

## Electronic supplementary material

Below is the link to the electronic supplementary material.

Supplementary material 1 (PDF 1180 kb)
