# Supplementary material for: Perfection of Perovskite Grain Boundary Passivation by Rhodium Incorporation for Efficient and Stable Solar Cells
Source: Nanomicro Lett. 2020 Jun 3;12:119. doi: 10.1007/s40820-020-00457-7 (PMC7770677; doi:10.1007/s40820-020-00457-7)
Supplement: Supplementary file 1 — Supplementary material 1 (PDF 1180 kb) [file 40820_2020_457_MOESM1_ESM.pdf]

Supplementary Information for

Perfection of Perovskite Grain Boundary Passivation by Rhodium Incorporation for Efficient and Stable Solar Cells

Wei Liu[1], Nanjing Liu[1], Shilei Ji[1], Hongfeng Hua[1], Yuhui Ma[1], Ruiyuan Hu[1], Jian Zhang[1], Liang Chu[1, 3] *, Xing'ao Li[1] *, Wei Huang[1, 2, *]

[1]Institute of Advanced Materials & New Energy Technology Engineering Laboratory of Jiangsu Province, Nanjing University of Posts and Telecommunications (NUPT), Nanjing 210023, People's Repbublic of China

[2]Shaanxi Institute of Flexible Electronics, Northwestern Polytechnical University, Xi'an 710072, People's Repbublic of China

[3]Guangdong Provincial Key Lab of Nano-Micro Materials Research, School of Chemical Biology and Biotechnology, Peking University Shenzhen Graduate School, Shenzhen 518055, People's Repbublic of China

*Corresponding authors. E-mail: chuliang@njupt.edu.cn (Liang Chu), iamxali@njupt.edu.cn (Xing'ao Li), iamwhuang@njupt.edu.cn (Wei Huang)

S1 Characterizations

The morphology of samples was acquired with scanning electron microscopy (SEM, Hitachi S-4800). X-ray diffraction (XRD) patterns were measured with Cu-Kα radiation (λ =1.5418 Å). Photoluminescence (PL) measurements were used by an Edinburgh F900. Time-resolved PL was measured by a FLS980E fluorometer (Edinburgh Instruments Ltd.), with an excitation wavelength of 785 nm. The UV-vis absorption spectra were recorded using a PerkinElmer Lambda 650S spectrophotometer with the wavelength range from 350 to 800 nm. The X-ray photoemission spectroscopy (XPS) and ultraviolet photoelectron spectroscopy (UPS) were measured using an AXIS Supra. The current density-voltage (*J*-*V*) characteristics of the PSCs were acquired by a Keithley 2400 Source under illumination of simulated sunlight (AM 1.5G, 100 mW/cm^2) provided by a solar simulator (Newport 91150 USA). Electrochemical impedance spectroscopy (EIS) and Mott–Schottky were measured by CHI660D electrochemical workstation.

Figure S8 shows the 1/C^2-V curves of the PSCs for determining the effective hole carrier concentration of the perovskite absorber layer. Using the Mott–Schottky method, the net carrier concentration can be evaluated based on the Equation (Eq. S1) [17]:

$$N_C(W) = \frac{2}{qK_S\varepsilon_0 A^2}\frac{d(\frac{1}{C^2})}{dV} \qquad (S1)$$

where $N_c(W)$ is the net carrier concentration, q is the electron charge, Ks is the semiconductor dielectric constant, ε_0 is the permittivity of free space, A is the area of the solar cell, C is the capacitance, and V is the applied voltage [17]. It demonstrates that the Rh^{3+}-doped perovskite devices have changed carrier concentration based on above equation, and they also have led to a higher built-in voltage. The higher built-in voltage is favorable for the improvement of open-circuit voltage (V_{oc}), which also accounts for the increase of V_{oc} for Rh^{3+}-doped perovskite device as shown in the measurements.

S2 Supplementary Figures and Tables

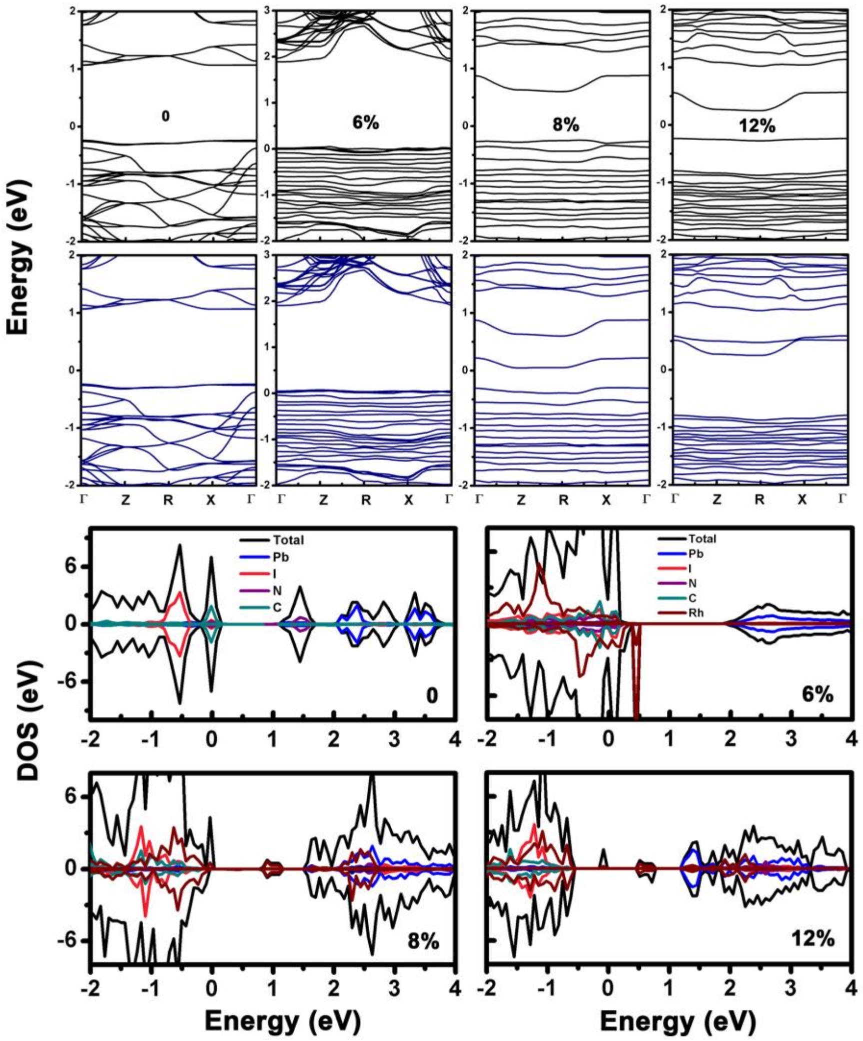

Fig. S1 Band structures, total density of states (DOS) and partial density of states (PDOS) of $MAPbI_3$:xRh (where x = 0, 6%, 8% and 12%)

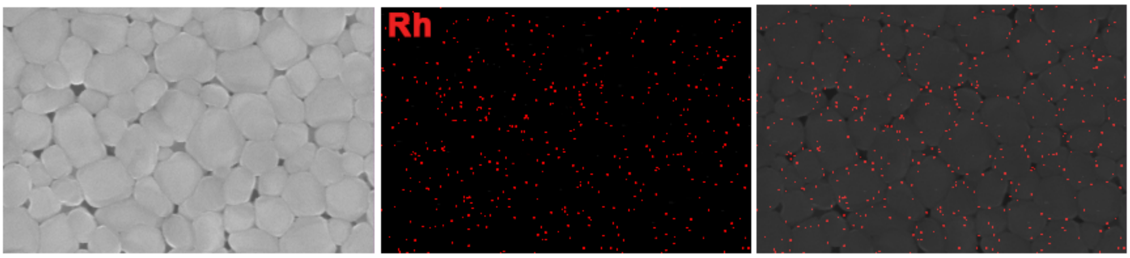

Fig. S2 EELS mapping distribution of $MAPbI_3$:xRh (where x = 1%)

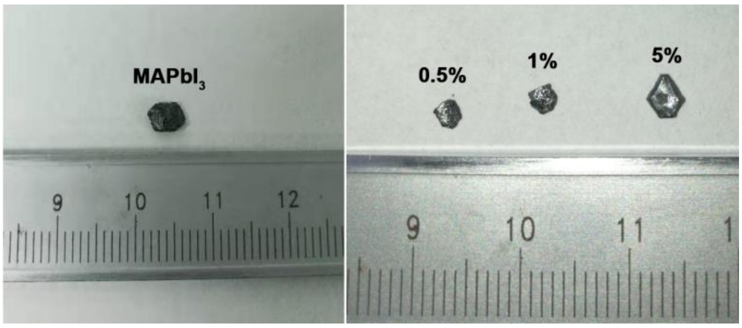

Fig. S3 Photos of the single crystalline perovskite $MAPbI_3$:xRh

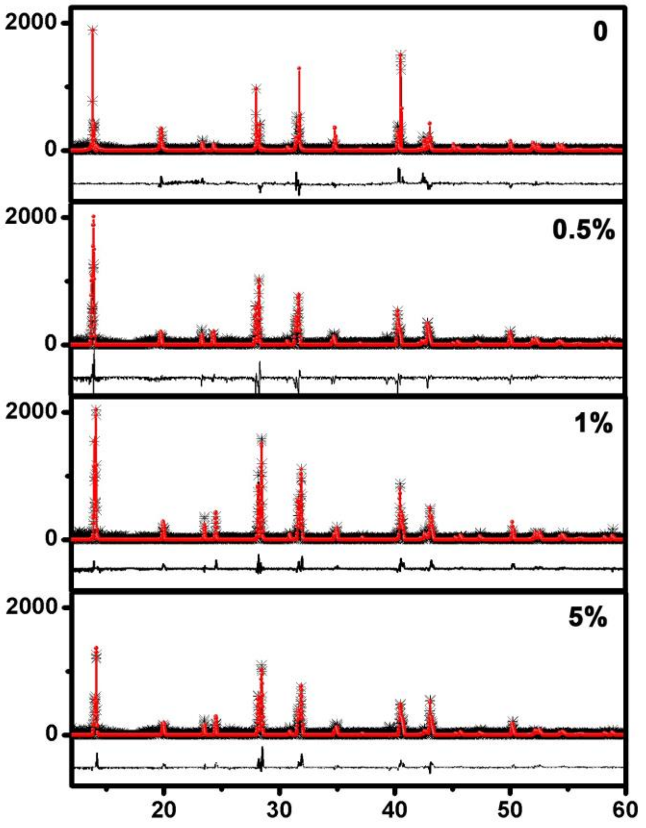

Fig. S4 Rietveld refinement of the XRD patterns of $MAPbI_3$:xRh (where x = 0, 0.5%, 1% and 5%). The differences between experiment (red circles) and calculated (black line) spectra are plotted at the bottom

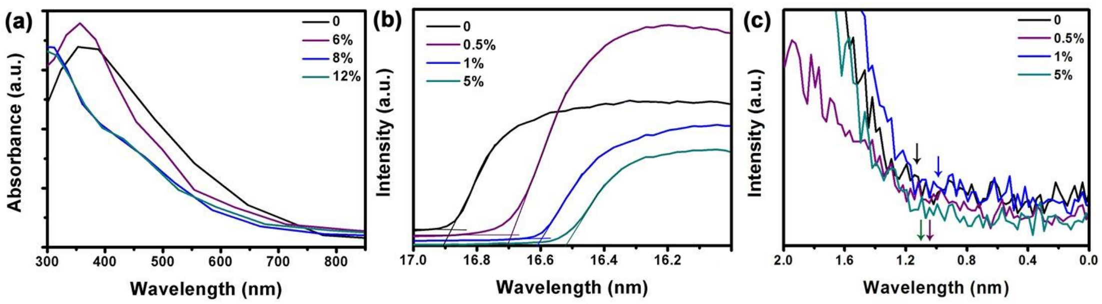

Fig. S5 a Absorbance coefficient of calculation results. **b** High binding energy and low binding energy **c** of perovskite films from UPS

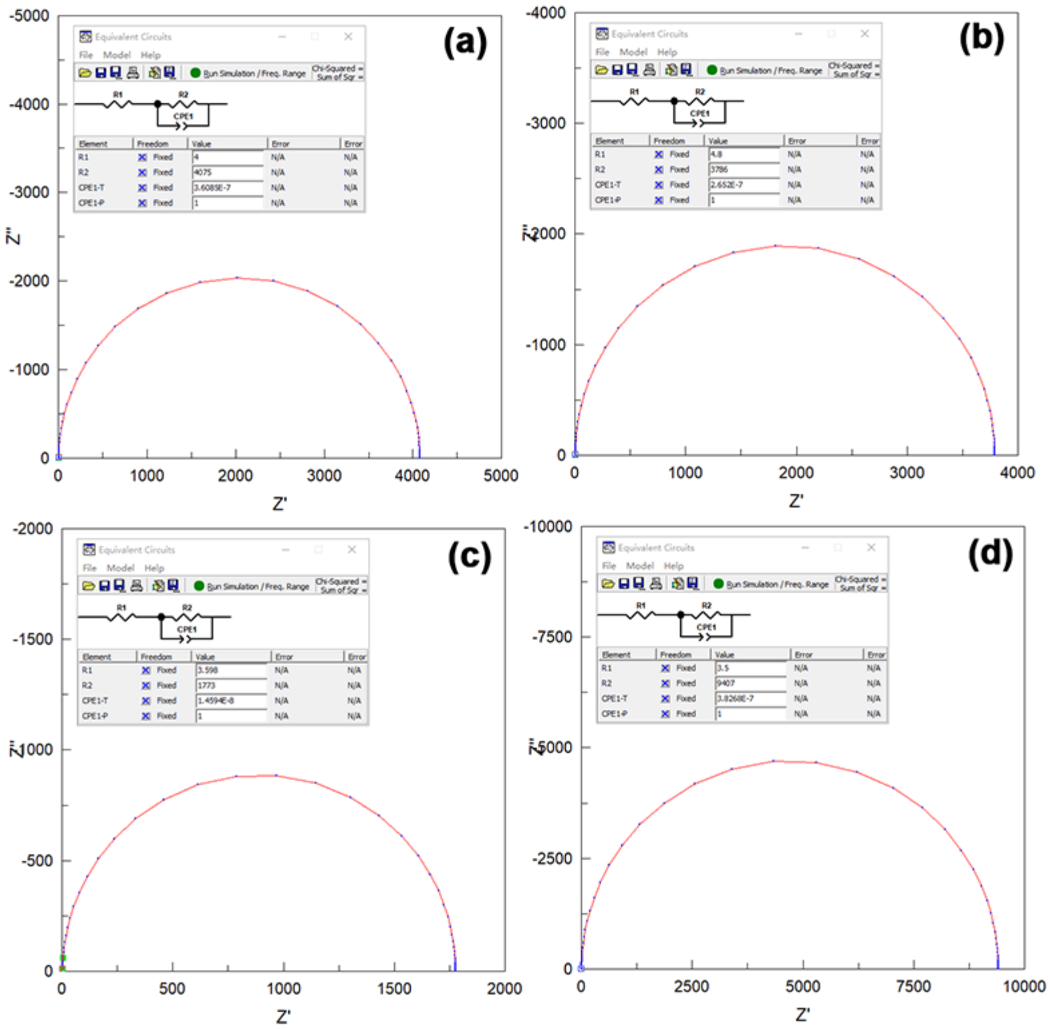

Fig. S6 Data-fitting result of Nyquist plots of PSCs based on $MAPbI_3$:xRh (where x = 0 **a**, 0.5% **b**, 1% **c**, and 5% **d**)

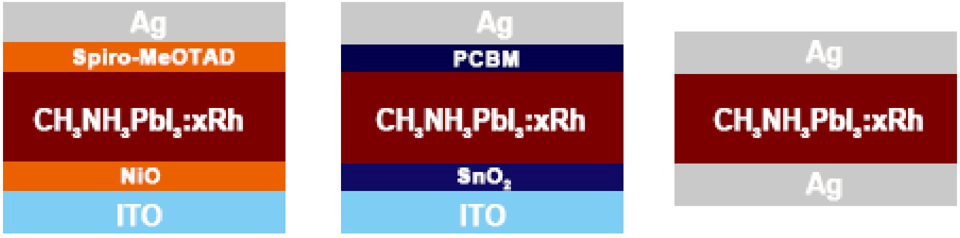

Fig. S7 Device architectures of the hole-only diode and, the electron-only diode perovskite-only device

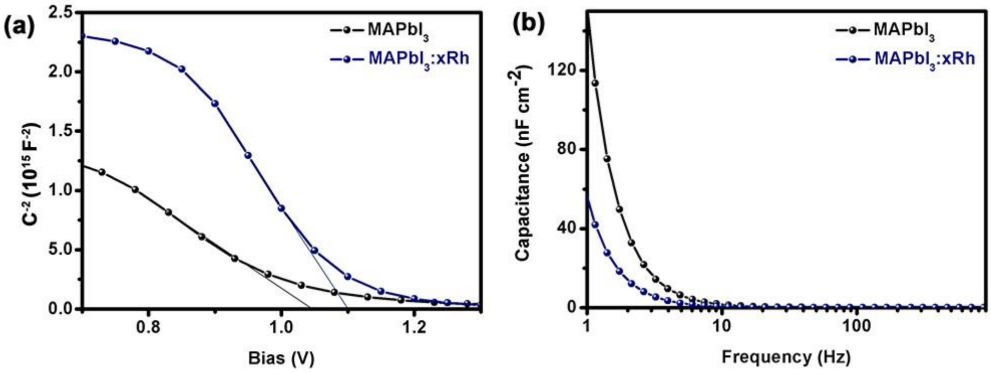

Fig. S8. a Mott-Schottky plot obtained from impedance measurements in dark. **b** Frequency-capacitance (F-C) curves measured from perovskite solar cells with and without 1% Rh^{3+} addition

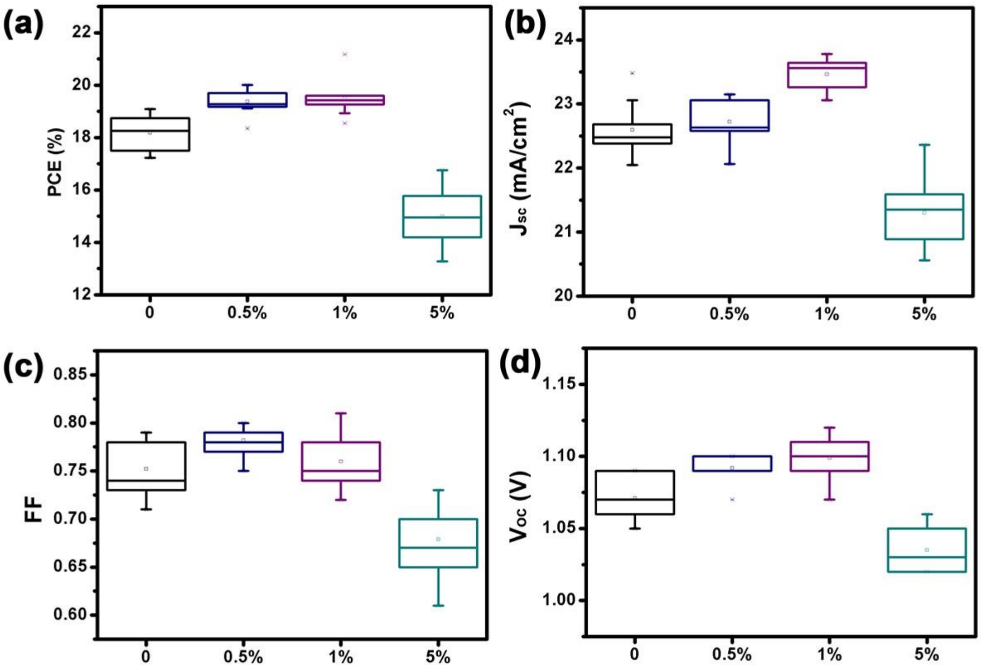

Fig. S9 Statistical photovoltaic parameters of the PSC based on $MAPbI_3$:xRh (where x = 0, 0.5%, 1%, and 5%). Photovoltaic parameters of **a** PCE, **b** *J*sc, **c** FF, and **d** V_{oc} coverage under simulated AM1.5 sunlight of 100 mW cm^{-2}. Each group includes 10 individual devices

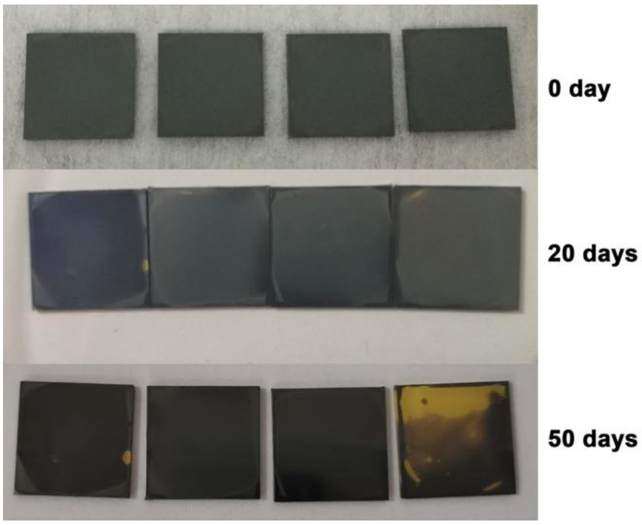

Fig. S10 Perovskite films based on $MAPbI_3$ and 1% Rh-doped are exposed in the air for 50 days

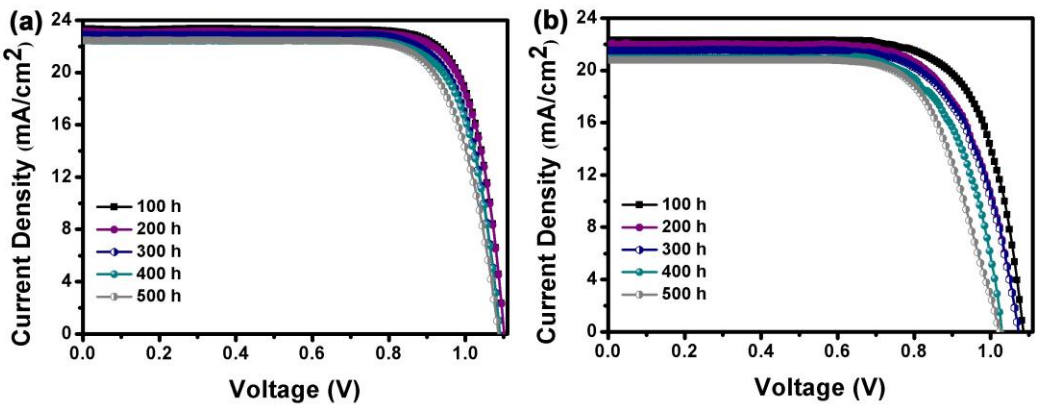

Fig. S11 J-V curves of PSCs based on $MAPbI_3$ and $MAPbI_3$:xRh with different time at dry air

Table S1 EELS mapping distribution of $MAPbI_3$:xRh (where x = 1%)

Element	Weight%	Atomic%
C	9.11	51.06
N	1.53	7.37
Rh	0.26	0.17
I	60.62	32.15
Pb	28.48	9.25
Totals	100.00	Rh/Pb = 1.8%

Table S2 Selected structural parameters from Rietveld refinement of $MAPbI_3$:xRh (where x = 0, 0.5%, 1%, and 5%)

Sample	$MAPbI_3$	0.5%	1%	5%	calculation
Cell type	I 4 c m	I 4 c m	I 4 c m	I 4 c m	I 4 c m
a	8.86812	8.87288	8.87044	8.86757	8.849
b	8.86812	8.87288	8.87044	8.86757	8.849
c	12.67568	12.68034	12.67468	12.66987	12.642
Cell volume (Å3)	996.8605035	998.2978011	997.3034668	996.2799945	989.929305
Pb-I bond distance (Å)	3.1538, 3.1689	3.15549, 3.17008	3.15463, 3.16847	3.15360, 3.16747	3.38899, 3.40495
Pb-I-Pb	89.987, 180	89.987, 180	89.987, 180	89.987, 180	
Rp, Rwp	7.55, 9.70	8.84, 11.63	9.13, 11.08	7.53, 9.66	

Table S3 Fermi energy, valence and conduction band from UPS and calculation

Rh-doped	high binding energy	Fermi energy (eV)	low binding energy	valence band (eV)	conduction band (eV)	experiment band gap (eV)	calculate band gap (eV)
0	16.89	-4.33	1.15	-5.48	-3.91	1.57	1.31
0.5%	16.70	-4.52	1.04	-5.56	-3.98	1.58	-
1%	16.60	-4.62	0.92	-5.54	-3.96	1.58	-
5%	16.52	-4.70	1.08	-5.78	-4.19	1.59	1.84

Table S4 Carrier lifetimes of $MAPbI_3$:xRh (where x = 0, 0.5%, 1%, and 5%) perovskite films

Sample	τ_1/ns	A_1/%	τ_2/ns	A_2/%	τ_{avg}/ns
$MAPbI_3$	67	0.2715	693	0.7285	671.229
0.5%	72	0.2878	751	0.7122	725.675
1%	63	0.2836	818	0.7164	795.662
5%	55	0.2985	677	0.7015	656.216

Table S5 Photovoltaic performance of PSCs based on $MAPbI_3$

No.	V_{oc} (V)	J_{sc} (mA/cm^2)	FF	PCE(%)
1	1.09	22.46	0.78	19.09
2	1.07	22.38	0.78	18.67
3	1.09	22.26	0.72	17.47
4	1.05	23.48	0.71	17.50
5	1.07	22.54	0.73	17.60
6	1.09	22.05	0.78	18.74
7	1.06	22.58	0.79	18.91
8	1.07	23.06	0.74	18.26
9	1.07	22.68	0.76	18.44
10	1.05	22.48	0.73	17.23
Average	1.07±0.02	22.60±0.8	0.75±0.04	18.19±0.96

Table S6 Photovoltaic performance of PSCs based on $MAPbI_3$:xRh (where x = 0.5%)

No.	V_{oc} (V)	J_{sc} (mA/cm^2)	FF	PCE(%)
1	1.09	23.13	0.79	20.01
2	1.10	22.06	0.80	19.41
3	1.09	22.58	0.78	19.20
4	1.09	23.06	0.79	19.86
5	1.09	22.68	0.80	19.70
6	1.07	23.15	0.79	19.64
7	1.10	22.59	0.78	19.28
8	1.09	22.45	0.75	18.35
9	1.10	22.89	0.77	19.17
10	1.10	22.63	0.77	19.12
Average	1.08±0.02	22.72±0.43	0.78±0.03	19.37±1.02

Table S7 Photovoltaic performance of PSCs based on $MAPbI_3$:xRh (where x = 1%)

No.	V_{oc} (V)	J_{sc} (mA/cm^2)	FF	PCE(%)
1	1.10	23.82	0.79	20.71
2	1.11	23.56	0.75	19.60
3	1.11	23.68	0.75	19.59
4	1.12	23.58	0.74	19.45
5	1.07	23.06	0.78	19.26
6	1.07	23.26	0.78	19.43
7	1.09	23.58	0.79	20.27
8	1.11	23.64	0.74	19.35
9	1.10	23.38	0.72	18.55
10	1.11	23.14	0.74	18.93
Average	1.10±0.03	23.47±0.41	0.76±0.05	19.51±1.2

Table S8 Photovoltaic performance of PSCs based on $MAPbI_3$:xRh (where x = 5%)

No.	V_{oc} (V)	J_{sc} (mA/cm^2)	FF	PCE(%)
1	1.06	21.44	0.73	16.76
2	1.02	21.35	0.61	13.28
3	1.04	20.56	0.65	13.90
4	1.03	21.68	0.67	14.96
5	1.03	20.89	0.67	14.42
6	1.05	21.47	0.63	14.20
7	1.05	20.58	0.73	15.77
8	1.03	21.06	0.70	15.18
9	1.02	21.59	0.70	15.41
10	1.02	22.36	0.70	15.96
Average	1.03±0.03	21.30±1.06	0.67±0.06	14.98±1.78

Table S9 Photocurrent hysteresis studies of the PSCs fabricated by pristine $MAPbI_3$ thin film and the $MAPbI_3$:xRh (x = 1 mol%) thin film

Devices		V_{oc} (V)	J_{sc} (mA/cm^2)	FF	PCE(%)	HI
$MAPbI_3$	Reverse	1.09	22.46	0.78	19.09	0.042
	Forward	1.09	21.76	0.77	18.26	
1% Rh	Reverse	1.10	23.82	0.79	20.71	0.020
	Forward	1.09	23.87	0.78	20.29	

Table S10 The hole and electron mobilities and the defect densities of pristine $MAPbI_3$ thin film and the $MAPbI_3$:xRh (x = 1%) thin film

Hybrid perovskite materials	Hole mobility (cm^2 V^{-1}s^{-1})	Electron mobility (cm^2 V^{-1}s^{-1})	Hole defects (cm^{-3})	Electron defects (cm^{-3})
$MAPbI_3$	1.29×10^{-3}	5.38×10^{-3}	3.10×10^{15}	2.74×10^{16}
$MAPbI_3$:xRh (x = 1%)	2.51×10^{-2}	1.26×10^{-2}	8.09×10^{15}	1.26×10^{15}

Table S11 Summary of the device performance data for PSCs based on different light absorbers and doping

Absorber	Doping	PCE(%)	Year	Refs.
MAPbI3	Al^{3+}	19.1%	2016	S1
MAPbI3	Li^{+}	18.1%	2017	S2
MAPbI3	Ag+	20.02%	2018	S3
MAPbI3	Zn^{2+}	18.35%	2018	S4
MAPbI3	Ni^{2+}	20.63%	2018	S5
MAPbI3	Mn^{2+}	19.09%	2019	S6
MAPbI3	R^{3+}	20.71%	-	This work
MAPbI3	Nd^{3+}	21.15%	2019	S7
$MAPb_{0.85}In_{0.15}I_3Cl_{0.15}$	In^{3+}	17.55%	2016	S8
$MAPbI_{3-x}Cl_x$	Ca^{2+}	12.0%	2017	S9
$MAPbI_{3-x}Cl_x$	Sr^{2+}	11.2%	2017	S9
$MAPbI_{3-x}Cl_x$	Ba^{2+}	14.9%	2017	S9
$MASr_aPb_{1-a}I_{3-x}Cl_x$	Sr^{2+}	16.3%	2017	S10
$MAPb_{0.9}Co_{0.1}I_3$	Co^{2+}	21.43%	2018	S11
$MAPb_{0.9}Sn_{0.05}Cu_{0.05}I_{2.9}Br_{0.1}$	Cu^{2+}	21.08%	2018	S12
$MAPb_xMg_{1-x}I_{3-y}Cl_y$	Mg^{2+}	14.2%	2018	S13
$MA_{1-x}K_xPbI_3$	K^{+}	11.05%	2019	S14
$FA_xMA_{1-x}PbI_3$	Eu^{3+}	21.52%	2019	S15
$(FA)_{0.83}(MA)_{0.17}PbI_{0.83}Br_{0.17}$	Gd^{3+}	21.21%	2020	S16

Table S12 Performance of PSCs based on $MAPbI_3$ and $MAPbI_3$:xRh with different time at dry air

No.	V_{oc} (V)	J_{sc} (mA cm^{-2})	FF	PCE(%)
$MAPbI_3$-100h	1.09	22.31	0.76	18.48
$MAPbI_3$-200h	1.07	21.94	0.71	16.67
$MAPbI_3$-300h	1.07	21.51	0.71	16.34
$MAPbI_3$-400h	1.03	20.91	0.71	15.29
$MAPbI_3$-500h	1.03	20.46	0.68	14.06
$MAPbI_3$:xRh-100h	1.10	23.36	0.79	20.30
$MAPbI_3$:xRh-200h	1.09	23.12	0.79	19.91
$MAPbI_3$:xRh-300h	1.09	22.92	0.78	19.49
$MAPbI_3$:xRh-400h	1.09	22.52	0.78	19.15
$MAPbI_3$:xRh-500h	1.08	22.45	0.77	19.04

Supplementary References

[S1]J.W. Wang, Z. Wang, S. Pathak, W. Zhang, D. W. deQuilettes, et al., Efficient perovskite solar cells by metal ion doping. Energy Environ. Sci. **9**(9), 2892-2901 (2016). https://doi.org/10.1039/C6EE01969B

[S2]J. Zhang, R. Chen, Y. Wu, M. Shang, Z. Zeng, Y. Zhang, Y. Zhu, L. Han, Extrinsic movable ions in $MAPbI_3$ modulate energy band alignment in perovskite solar cells. Adv. Energy Mater. **8**(5), 1701981 (2018). https://doi.org/10.1002/aenm.201701981

[S3]Y. Zhang, C.C. Zhang, C.H. Gao, M. Li, X. J. Ma, et al., N-type doping of organic-inorganic hybrid perovskites toward high-performance photovoltaic devices. Solar RRL **3**(2), 1800269 (2019). https://doi.org/10.1002/solr.201800269

[S4]H. Zheng, G. Liu, X. Xu, A. Alsaedi, T. Hayat, X. Pan, S. Dai, Acquiring High-Performance and Stable Mixed-Dimensional Perovskite Solar Cells by Using Transition Metal Substituted Pb. ChemSusChem **11**, 3269-3275 (2018). https://doi.org/10.1002/cssc.201801171

[S5]X. Gong, L. Guan, H. Pan, Q. Sun, X. Zhao, et al., Highly efficient perovskite solar cells via nickel passivation. Adv. Funct. Mater. **28**(50), 1804286 (2018). https://doi.org/10.1002/adfm.201804286

[S6]W. Liu, L. Chu, N. Liu, Y. Ma, R. Hu, et al., Efficient perovskite solar cells fabricated by manganese cations incorporated in hybrid perovskites. J. Mater.

Chem. C **7**(38), 11943-11952 (2019). https://doi.org/10.1039/C9TC03375K

[S7] K. Wang, L. Zheng, T. Zhu, X. Yao, C. Yi, et al., Efficient perovskite solar cells by hybrid perovskites incorporated with heterovalent neodymium cations. Nano Energy **61**, 352-360 (2019). https://doi.org/10.1016/j.nanoen.2019.04.073

[S8] Z. K. Wang, M. Li, Y. G. Yang, Y. Hu, H. Ma, et al., High efficiency Pb-In binary metal perovskite solar cells. Adv. Mater. **28**(31), 6695-6703 (2016). https://doi.org/10.1002/adma.201600626

[S9] S.H. Chan, M.C. Wu, K.M. Lee, W.C. Chen, T.H. Lin, W.F. Su, Enhancing perovskite solar cell performance and stability by doping barium in methylammonium lead halide J. Mater. Chem. A **5**, 18044-18052 (2017). https://doi.org/10.1039/C7TA05720B

[S10] X. Shai, L. Zuo, P. Sun, P. Liao, W. Huang et al., Efficient planar perovskite solar cells using halide Sr-substituted Pb perovskite. Nano Energy **36**, 213-222 (2017). https://doi.org/10.1016/j.nanoen.2017.04.047

[S11] W. Xu, L. Zheng, X. Zhang, Y. Cao, T. Meng et al., Efficient perovskite solar cells fabricated by Co partially substituted hybrid perovskite. Adv. Energy Mater. **8**(14), 1703178 (2018). https://doi.org/10.1002/aenm.201703178

[S12] M. Li, Z.K. Wang, M.P. Zhuo, Y. Hu, K.H. Hu et al., Pb–Sn–Cu ternary organometallic halide perovskite solar cells. Adv. Mater. **30**, 1800258 (2018). https://doi.org/10.1002/adma.201800258

[S13] P. Singh, P. J. S. Rana, R. Mukherjee, P. Srivastava, A step towards environmental benign Mg/Pb based binary metal mixed halide perovskite material Sol. Energy **170**, 769-779 (2018). https://doi.org/10.1016/j.solener.2018.05.090

[S14] Y. Yao, X. Zou, J. Cheng, T. Ling, C. Chang, D. Chen, Impact of K^+ doping on modulating majority charge carrier type and quality of perovskite thin films by two-step solution method for solar cells. Coatings **9**, 647 (2019). https://doi.org/10.3390/coatings9100647

[S15] L. Wang, H. Zhou, J. Hu, B. Huang, M. Sun, et al., A Eu^{3+}-Eu^{2+} ion redox shuttle imparts operational durability to Pb-I perovskite solar cells. Science **363**(6424), 265-270 (2019). https://doi.org/10.1126/science.aau5701

[S16] Y. Yang, J. Wu, X. Wang, Q. Guo, X. Liu et al., Suppressing vacancy defects and grain boundaries via ostwald ripening for high−performance and stable perovskite solar cells**.** Adv. Mater. 1904347 (2019). https://doi.org/10.1002/adma.201904347

[S17] W. Zhao, Z. Yao, F. Yu, D. Yang, S.F. Liu, Alkali metal doping for improved $CH_3NH_3PbI_3$ perovskite solar cells. Adv. Sci. **5**(2), 1700131 (2018). https://doi.org/10.1002/advs.201700131
